# Does Intent Regarding Abusive Supervision Really Matter? The Moderating Effect of Performance-Promotion and Injury-Initiation Attributions Between Abusive Supervision and Emotional Exhaustion

**DOI:** 10.3390/bs16030444

**Published:** 2026-03-18

**Authors:** Teng Liu, Steven Kilroy, Yan Zhang

**Affiliations:** 1 Trinity Business School, Trinity College Dublin, D02 F6N2 Dublin, Ireland; 2 School of Psychological and Cognitive Sciences, Peking University, Beijing 100871, China

**Keywords:** abusive supervision, performance-promotion attribution, injury-initiation attribution, emotional exhaustion, attribution theory, conservation of resources theory

## Abstract

While prior research shows that subordinates’ attributions can amplify or buffer the negative effects of abusive supervision on performance outcomes, it remains unclear whether similar moderating effects extend to subordinate well-being. Drawing on attribution theory and conservation of resources (COR) theory, this study investigates whether performance-promotion and injury-initiation attributions moderate the relationship between abusive supervision and emotional exhaustion. Applying a time-lagged research design, we surveyed full-time employees (N = 224) within a single Chinese transportation company and tested the proposed hypotheses using structural equation modeling (SEM). Contrary to the expectations and prior evidence, the moderating effect of injury-initiation attribution between abusive supervision and emotional exhaustion is nonsignificant. Moreover, performance-promotion attribution significantly moderates this relationship, in the opposite direction to the expectations: It exacerbates (rather than buffers) the positive association between abusive supervision and emotional exhaustion. These findings complicate the assumption that performance-promotion attributions are protective whereas injury-initiation attributions are destructive, instead suggesting a different pattern of attributional effects. The study advances the understanding of abusive supervision attributions and provides implications for management practice.

## 1. Introduction

Tepper (2000, p. 178) defined abusive supervision as employee perceptions of “the sustained display of hostile, verbal and nonverbal behaviors, excluding physical contact” from their supervisors. A substantial body of literature has shown that abusive supervision can lead to subordinates’ psychological harm (e.g., stress, anger, and alienation) and deviant behaviors, such as retaliation and counterproductive work behaviors (see Fischer et al., 2021; Tepper et al., 2017, for reviews). While prior studies have extended our understanding of the negative consequences of abusive supervision and what may cause it, there remains a lacuna of research on potent coping strategies to grapple with the detrimental effects of abusive supervision (Tepper et al., 2017). Understanding how to restrain the influence of abusive supervision is critical since it may be myopic to believe that supervisory abusive behaviors in the workplace can be fully eradicated (Fischer et al., 2021).

Drawing on attribution theory (Heider, 1958), the influence of abusive supervision is conditional (Fischer et al., 2021; Y. Liang et al., 2024; Tepper et al., 2017) and highly dependent on subordinates’ attributions (e.g., Liu et al., 2012; Yu & Duffy, 2021). Liu et al. (2012) conceptualized two distinct motives underlying leaders’ abusive behaviors: Performance-promotion, reflecting intent to enhance subordinates’ performance, and injury-initiation, reflecting intent to deliberately harm subordinates. Subsequent evidence has found support for the moderating effects of subordinate attributions, such that abused subordinates who attribute abusive supervision to performance-promotion could effectively subdue the negative influence of abusive supervision, while the negative impact for those who perceive injury-initiation attributions could be amplified (Huh & Lee, 2022; Kim et al., 2019; Yu & Duffy, 2021). While extant studies have discovered that individual attributions can either alleviate or exacerbate the negative effects of abusive supervision on subordinates’ performance outcomes, including creativity (Liu et al., 2012), job performance (Yang et al., 2020), and OCBs (Yu & Duffy, 2021), whether similar moderating effects extend to subordinate well-being remains underexplored. Addressing this void is crucial because, without protecting well-being, the maintained performance and efficiency would likely be short-lived and thus not result in a sustainable solution.

Based on conservation of resource (COR) theory (Hobfoll, 1989) and prior empirical evidence (e.g., Huh & Lee, 2022; Kim et al., 2019; Yu & Duffy, 2021), construing abusive supervision as performance-promotion provides an instrumental cognitive resource that values goal-related meaning and facilitates coping, thereby offsetting resource loss and mitigating emotional exhaustion. However, the beneficial aspects of positive attribution (or low negative attribution) on abused subordinates’ performance might not necessarily reflect on their well-being. Studies have suggested that performance improvements sometimes stem from subordinates’ intensified effort and increased work investment at a cost to their well-being (Hunter & Thatcher, 2007; LePine et al., 2005). Importantly, sustainable performance is more likely when employees’ well-being is protected, rather than when performance is maintained through continuous resource depletion (Yang et al., 2020; Yu & Duffy, 2021). Thus, the present study aims to examine whether abused subordinates with performance-promotion attribution also experience lower levels of emotional exhaustion, or in the opposite way.

**Figure 1:**
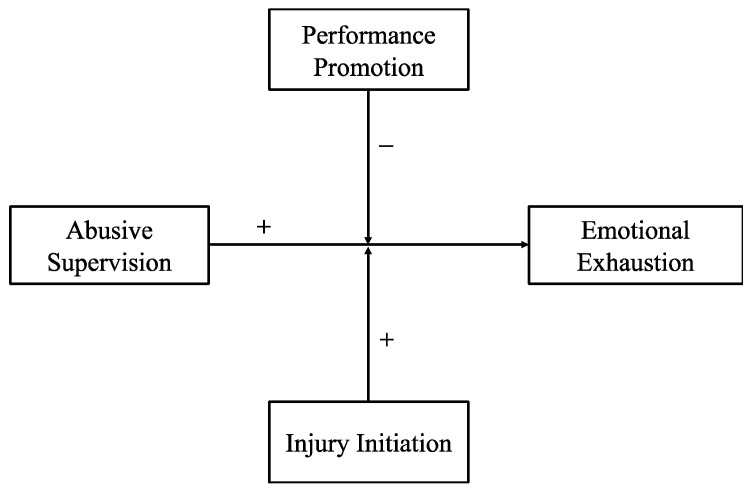
Conceptual Framework.

Likewise, it is meaningful to delve into the moderating effect of injury-initiation attribution on the relationship between abusive supervision and individual well-being. Drawing on attribution theory (Heider, 1958), perceiving abusive supervision as intentionally harmful (e.g., destructive leader) may significantly amplify psychological distress, leading to severe emotional exhaustion. Past literature also documented the exacerbating effects of injury-initiation attribution on the influence of abusive supervision on performance (Liu et al., 2012; Yu & Duffy, 2021). However, there is dearth of empirical evidence to support that such exacerbating effects extend to well-being outcomes. Importantly, another perspective inherent to attribution theory implies this moderation may be less pronounced than expected, because perceiving abusive supervision negatively may be intrinsic to abusive encounters and thus exert minimal incremental influence beyond the main effect (Kelley, 1973). In this regard, the moderating effect of injury-initiation attribution between abusive supervision and well-being might be suspectable. To understand the above questions, the present study employs attribution theory (Heider, 1958) and conservation of resources (COR) theory (Hobfoll, 1989) to investigate whether and how performance-promotion and injury-initiation attributions moderate the association between abusive supervision and subordinates’ emotional exhaustion (see Figure 1).

The study offers two primary contributions. First, although performance-promotion attribution enables subordinates to sustain performance outcomes (e.g., Huh & Lee, 2022; Kim et al., 2019; Yu & Duffy, 2021), its protective value for well-being among abused subordinates remains uncertain. Based on COR theory and attribution theory, the present study seeks to address the void by examining whether the beneficial aspect of performance-promotion attribution also materializes for employee well-being (i.e., emotional exhaustion). In doing so, the study investigates whether the positive effects of performance-promotion attribution reflect subordinates’ genuine well-being improvement. In the same vein, scholars have discovered that injury-initiation attribution can exacerbate the negative impact of abusive supervision on subordinates’ work performance (Liu et al., 2012; Yu & Duffy, 2021). However, the empirical evidence remains sparce regarding whether this exacerbating effect extends to well-being outcomes. Our study addresses this void by examining whether injury-initiation attribution moderates the relationship between abusive supervision and subordinates’ emotional exhaustion. Secondly, due to the prevalence of the abusive supervision phenomenon in China and the broader Asian context (L. H. Liang et al., 2016; Mackey et al., 2017; Wang et al., 2020), our study represents an important contribution by testing our proposed model using multi-time data collected in a single company in China where coping strategies against abusive leaders are urgently needed (L. H. Liang et al., 2016; Mackey et al., 2017). The findings provide important recommendations for management practice in a context in which abusive supervision is unlikely to dissipate any time soon.

## 2. Literature Development

Substantial evidence demonstrates that abusive supervision is associated with a range of adverse outcomes and considerable organizational costs, such as higher levels of subordinates’ absenteeism, increased health-care cost, lost productivity, and high turnover (Schat et al., 2006). Abusive supervision is acknowledged to be particularly prevalent in high-power-distance Asian contexts where supervisory abusive behaviors are often less criticized and punished (Mackey et al., 2017). Evidence has shown that more than 50% of Chinese employees experience supervisory abuse and cold violence (L. H. Liang et al., 2016; Wang et al., 2020). While understanding coping strategies against abusive supervision is urgently needed, there is a lacuna of knowledge regarding how to restrain and counteract the detrimental effects of abusive supervision (Fischer et al., 2021; Tepper et al., 2017).

One research stream has suggested that the negative impact of abusive supervision on organizational outcomes is not inevitable (Fischer et al., 2021; Y. Liang et al., 2024; J. Zhang & Liu, 2018). In practice, abusive supervision could stimulate subordinates’ performance via a performance enhancement pathway, including attention, desire to avoid further hostility, and prove the supervisor wrong (Tepper, 2007; Tepper et al., 2017). Moreover, a variety of factors may prevent or perpetuate the impacts of this leadership style on employees, as reflected by the substantial variability in reported associations between abusive supervision and employee deviance, ranging from as low as −0.15 to as high as 0.74 (Fischer et al., 2021). In this regard, it is worthwhile to uncover the boundary factors which may alter the serious detriments of abusive supervision into trivial effects. Based on this knowledge, practitioners will be better equipped to introduce pertinent management strategies to grapple with abusive supervision in practice.

### 2.1. Abusive Supervision and Attribution Theory

A promising strand of research underscores the value of an attributional perspective for deepening the contemporary understanding of why the effects of abusive supervision could vary (Liu et al., 2012; Martinko et al., 2011). According to attribution theory (Heider, 1958), people construct causal explanations for social stimuli, which in turn influence their emotions and guide their attitudinal and behavioral reactions (Heider, 1958). By applying the rationale to abusive supervision research, employees’ psychological response to abusive supervision may highly depend on how they attribute supervisory abusive behaviors (Liu et al., 2012). Indeed, although abusive supervision is commonly considered under a default expectation of supervisors’ deleterious motives (e.g., threatening or hurting subordinates), the measurement of abusive supervision captures neither leaders’ motives nor subordinates’ attributions of abusive behaviors (Fischer et al., 2021).

Initially, Tepper (2000) posited that leaders’ abusive behaviors could be enacted as part of a strategic management approach, for instance, to signal that mistakes or slackness would not be tolerated. Extending this perspective, Tepper (2007) further suggested that abusive supervision might be constructive when such aggressive behaviors were not perceived as intentionally harmful. Following this research line, Liu et al. (2012) introduced the notion that supervisors could exercise abusive behaviors for the purposes of (1) performance-promotion or (2) injury-initiation. Their empirical study revealed that the attributions made by employees could effectively buffer the negative relationship between abusive supervision and creativity (Liu et al., 2012). Based on Liu et al.’s study (Liu et al., 2012), Yang et al. (2020) found that abusive supervision was positively associated with job performance via performance-promotion attribution. In addition, Yu and Duffy (2021) discovered that viewing abusive supervision as performance-promotion intent elicited guilt, increasing OCB, whereas injury-initiation attribution elicited anger, thereby increasing deviance. Furthermore, Huh and Lee (2022) found that when employees made performance-promotion attributions, the detrimental effect of abusive supervision on work engagement became less harmful and could even appear beneficial. While these studies have identified managing attributions as effective aspects to alleviate the detriments of abusive supervision on their working performance, it remains unclear whether similar moderating patterns extend to subordinates’ health-related well-being. Addressing this void is important, because performance maintenance led by high-level performance-promotion attribution or low-level injury-initiation attribution could reflect genuine well-being and working experience improvement, but also might potentially rely on emotional regulation and resource depletion, i.e., impaired well-being (Hunter & Thatcher, 2007; LePine et al., 2005).

### 2.2. Abusive Supervision, Attributions, and Emotional Exhaustion

Conservation of resources (COR) theory (Hobfoll, 1989) indicates that individuals strive to protect their current resources and pursue new resources, and stress occurs when they experience a threat or the actual loss of resources (e.g., excessive demands). In accordance with COR theory, abusive supervision has been regarded as a critical job demand which drains subordinates’ resources, resulting in poor well-being (e.g., Aryee et al., 2008; L. H. Liang et al., 2018; Wu & Hu, 2009). In the present study, we focus on emotional exhaustion as one of the most recognized psychological symptoms associated with abusive supervision. According to the World Health Organization’s (WHO) International Classification of Diseases (ICD-11), the description of burnout, which is based on the definition and measure developed by Maslach and colleagues (Maslach et al., 1996), i.e., the Maslach Burnout Inventory (MBI), is defined as an occupational phenomenon resulting from chronic workplace stress that has not been successfully managed (World Health Organization, 2019). Emotional exhaustion represents the core component of burnout, referring to a state of physical and mental exhaustion caused by excessive stress (Maslach et al., 1996). Emotional exhaustion is a detrimental outcome that jeopardizes individuals’ capacity to perform at work (Cropanzano et al., 2003).

Meta-analytic evidence from more than 100 samples showed a moderate-to-strong positive association between abusive supervision and emotional exhaustion (Mackey et al., 2017). Additionally, longitudinal field research revealed that exposure to abusive supervision predicted subsequent elevated exhaustion after controlling for the possibility of reverse causality (L. H. Liang et al., 2018). These findings are in accordance with the central tenets of COR theory, whereby abusive supervision can persistently erode subordinates’ emotional and physical resources, essentially resulting in emotional exhaustion (Wu & Hu, 2009). Based on this coherent theoretical perspective and empirical evidence, we predict the following hypothesis:

**H1:**  *Abusive supervision will be positively related to subordinates’ individual emotional exhaustion.*

### 2.3. The Moderating Effect of Performance-Promotion Attribution

In practice, leaders’ abusive behaviors might be conducive to subordinates’ performance via performance-enhancement pathways (Tepper, 2007). For instance, leaders may sometimes resort to harsh language or aggressive gestures to correct or halt clearly inappropriate behaviors. Thus, aggressive words and deeds might be occasionally conducted to stimulate better performance rather than in the quest to cause harm (Liu et al., 2012; Yang et al., 2020; Yu & Duffy, 2021). As the purpose is aligned with their own benefit, subordinates could perceive leaders’ aggressive behaviors as a form of “tough love” containing information to be seen as making corrections and communicating high expectations (Liu et al., 2012). Additionally, this meaning-making may be particularly plausible in certain workplaces (e.g., eastern Asia), which are often characterized by relatively high-power-distance and hierarchical role norms that largely legitimize supervisory authority (House et al., 2004). In such a cultural context, subordinates may be more likely to construe harsh supervision as discipline or performance management (rather than interpersonal hostility), making performance-promotion attribution especially salient for subsequent appraisals and coping. Based on existing evidence (Liu et al., 2012; Yu & Duffy, 2021), the legitimization of leaders’ aggressiveness generally positions performance-promotion attribution as an individual resource, encouraging subordinates to prevent further resource losses and maintain their performance (e.g., committing significant errors and receiving any resulting penalties).

However, from a competing perspective, performance-promotion attribution may simultaneously legitimize harsh treatment as “necessary for results,” which can heighten performance pressure, internalize self-blame, and facilitate persistence under demands (Hunter & Thatcher, 2007; LePine et al., 2005). Drawing on COR theory, performance-promotion attribution may trigger continued resource investment (e.g., sustained self-regulation and vigilance) to enhance performance, thereby potentially accelerating resource depletion and exacerbating strain. Accordingly, performance-promotion attribution could either attenuate or intensify the harmful impact of abusive supervision, depending on which process dominates. Aligning with prior evidence (e.g., Huh & Lee, 2022; Liu et al., 2012; Yu & Duffy, 2021), the buffering pathway is expected to dominate overall because performance-promotion attribution generally reframes leaders’ aggressiveness as performance-oriented “tough love”, which is associated with less downstream harm.

Specifically, when subordinates interpret leaders’ abusive behaviors as performance-promotion, three COR-relevant mechanisms could unfold. First, the primary appraisal shifts from a pure threat to a combination of warning and encouragement (Liu et al., 2012). Although the tone is harsh, the ultimate goal of improving one’s performance implies a potential resource gain (e.g., future mastery, recognition, or rewards). COR theory holds that the prospect of resource acquisition can offset current resource loss, thereby preventing emotional exhaustion (Hobfoll, 1989). Second, the stress caused by abusive supervision could be alleviated when subordinates reinterpret the supervisor’s intent from malevolent to constructive. Drawing on attribution theory (Heider, 1958), destructive intent elicits anger and moral outrage, thereby taxing self-regulatory resources and accelerating exhaustion (Yu & Duffy, 2021). In contrast, an instrumental intent tends to evoke concern for self-improvement. More importantly, abused subordinates could realize that the leader’s initial intent behind abusive behaviors is highly related to their shortcomings, consistent with their career development, and ultimately intended to benefit them (Yu & Duffy, 2021). Though these abusive behaviors are unethical and uncomfortable, they motivate constructive coping strategies and positive performance, which is less energetically costly than anger-fueled rumination or covert retaliation (Hobfoll et al., 2018). Third, performance-promotion attribution preserves the social resource of leader legitimacy. Believing that the supervisor’s harshness serves a developmental purpose sustains a baseline of trust and respect in the leader–member relationship, which can keep open channels for support and guidance that would otherwise be severed. This continuity mitigates the social isolation and identity threat that drive resource loss under abusive supervision.

In conclusion, performance-promotion attribution could buffer the relationship between abusive supervision and emotional exhaustion, because it potentially (1) introduces anticipated resource gains in the future, (2) substitutes positive essence for the damaging behaviors, and (3) safeguards relational resources with leaders. Taken together, even though abused subordinates endure the same abusive acts and often invest extra effort and resources to enhance their performance, these processes help conserve personal resources and shield them from elevated emotional exhaustion. Following previous research underscoring the buffering role of performance-promotion attribution in the negative influence of abusive supervision (e.g., Huh & Lee, 2022; Kim et al., 2019; Yang et al., 2020), we advance the following hypothesis:

**H2:**  *Performance-promotion attribution moderates the positive relationship between abusive supervision and individual emotional exhaustion, such that the relationship is weaker when performance-promotion attribution is higher (vs. lower).*

### 2.4. The Moderating Effect of Injury-Initiation Attribution

On the contrary, when subordinates construe the same leaders’ abusive behaviors as injury-initiation, the appraisal pivots from a performance enhancement to an unambiguous threat (Liu et al., 2012). Prior evidence has shown injury-initiation attribution can exacerbate the detrimental impact of abusive supervision, diminishing employees’ creativity (Liu et al., 2012) and heightening supervisor-directed deviance (Yu & Duffy, 2021). According to COR theory, this hostile attribution could undermine the possible developmental value of positive attributions and is likely to precipitate further resource loss, exacerbating emotional exhaustion.

Drawing on attribution theory (Heider, 1958), when subordinates attribute abusive supervision as injury-initiation, the primary appraisal tends to become threat- and harm-oriented with little compensatory gain prospects. Perceiving malicious intent precludes construing abusive supervisory behaviors as instrumentally motivated (e.g., performance orientation), leading employees to code them as intentional harm (Liu et al., 2012). Unlike challenge appraisals, which can convert some resource expenditure into future returns or performance improvement, a pure-threat schema offers little offsetting gain and thus accelerates resource depletion. In addition, the malevolence of injury-initiation attribution elicits negative emotions including anger, moral outrage, and desires for retribution (Yu & Duffy, 2021), all of which are physically and psychologically taxing. Based on COR theory, the energy devoted to managing anger, plotting retaliation, or suppressing public hostility may lead to serious resource loss, resulting in emotional exhaustion (Hobfoll et al., 2018). Moreover, injury-initiation attribution could damage the relationship between leaders and subordinates, making abusive leaders’ credibility collapse, along with the expectation of future support, mentorship, and fair treatment (Liu et al., 2012). In this manner, abused subordinates suffer further social resource depletion over time; therefore, viewing abusive supervision through an injury-initiation lens could seriously intensify emotional exhaustion. Nevertheless, it is also worth noting that the strength of this exacerbating effect might not necessarily be uniform across situations, because the salience and mechanisms of injury-initiation intent can vary across contexts and relationships. Particularly, the incremental role of injury-initiation attribution may be constrained in settings where the leader’s negative intent is already a default inference (Kelley, 1973), which could limit the incremental strength of its moderating effect even if the direction remains exacerbating. However, consistent with the predominant evidence that injury-initiation attributions intensify detrimental reactions (Liu et al., 2012; Yu & Duffy, 2021), we nonetheless propose the following hypothesis:

**H3:**  *Injury-initiation attribution moderates the positive relationship between abusive supervision and individual emotional exhaustion, such that the relationship is stronger when injury-initiation attribution is higher (vs. lower).*

## 3. Methods

### 3.1. Sample and Procedure

We collected survey data across two waves from a large transportation company in Southwest China. The organization’s core business involves highway and railway construction and road-related services. With support from the HR department, surveys were distributed to all full-time and on-payroll employees across major departments (e.g., marketing, maintenance engineering, operational safety, and accounting). Restricting the sampling frame to formally employed staff helps ensure that participants have relatively stable job roles and sustained interaction with their immediate supervisors, which is important for reliably assessing supervisory behaviors and psychological states (attributions and emotional exhaustion). All participants had an identifiable direct supervisor. Prior to the main study, we conducted a pilot test to ensure item clarity and readability. Following this, HR managers helped to distribute the survey links via WeChat (the most popular social media platform in China). The cover page of the two-wave survey stated that participation was voluntary, and that confidentiality was strictly maintained.

This design represents a single-organization, census-based, multi-wave sampling approach (i.e., surveying all eligible employees) enabled by organizational access. Collecting data within one organization helps minimize heterogeneity arising from external contextual differences (e.g., HR systems and industry/regional practices), which is especially important for multilevel modeling because it reduces the influence of exogenous between-organization variance. In addition, the two-wave design with predictors and outcomes measured at different time points helps reduce concerns about common method bias and provides a more conservative test of the hypothesized relationships.

At Time 1, employees rated abusive supervision, performance-promotion and injury-initiation attributions, and demographic information. At Time 2 (two weeks later), employees rated emotional exhaustion. A total of 280 staff members were eligible to participate in the survey. At Time 1, 258 subordinates participated (response rate of 92.14%). At Time 2, 252 participated (response rate of 89.64%). Following procedures to identify careless responders (Meade & Craig, 2012), 11 participants were excluded according to the careless check items (e.g., uniformly choosing “5” and repeating “123456”). By matching the multi-time surveys, the final multi-level sample was 224 participants from 37 groups (average group size = 6.05), leading to a response rate of 80%. Among the participants, 155 (69.2%) were male and 69 (30.8%) were female; their average age was 42.93 years (SD = 8.97); the mean tenure was 9.98 years (SD = 8.62).

### 3.2. Variable Measurement

Following Brislin’s (1980) procedures of back translation, we translated the surveys from English into Chinese and adjusted the items according to the firm context. All the measurement items are presented in Appendix A.

Abusive supervision. Tepper’s (2000) 15-item scale was used to measure abusive supervision. Employees answered items on a 5-point Likert scale, ranging from 1 (never happens to me) to 5 (he/she always does this to me). A sample item is “My supervisor gives me the silent treatment”. The Cronbach’s alpha was 0.97.

Performance-promotion attribution. Performance-promotion attribution was measured using five items representing the performance-promotion dimension of Liu et al.’s (2012) 10-item abusive supervision attribution scale. Before responding, participants were presented with the definition of abusive supervision and then rated each item on a 6-point Likert scale indicating the extent to which they agreed that each statement reflected the reason/intent underlying their supervisor’s abusive behaviors. A sample item is “Desire to elicit high performance from me”. The Cronbach’s alpha was 0.89.

Injury-initiation attribution. Injury-initiation attribution was measured using five items representing the injury-initiation dimension of Liu et al.’s (2012) 10-item abusive supervision attribution scale. Before responding, participants were presented with the definition of abusive supervision and then rated each item on a 6-point Likert scale indicating the extent to which they agreed that each statement reflected the reason/intent underlying their supervisor’s abusive behaviors. A sample item is “Desire to cause injury to me”. Cronbach’s alpha was 0.91.

Emotional exhaustion. Emotional exhaustion was measured with the five items that capture this dimension from Maslach et al.’s (1996) burnout scale. Participants answered a 6-point frequency scale ranging from 1 (never) to 6 (every day) A sample items is “I feel emotionally drained from my work”. The Cronbach’s alpha was 0.93.

Demographic and Control Variables. Following Y. Zhang and Bednall’s (2016) study, we controlled for gender (1 = male, 2 = female), age (years), and time working with the supervisor (years), as meta-analytic evidence suggests that demographic characteristics may show small but systematic associations with perceptions of abusive supervision. Prior literature has highlighted that leaders’ behavioral patterns might vary based on subordinates’ gender and age, as well as time working with the supervisor (See Y. Zhang & Bednall, 2016, for a review). By controlling for these individual demographic variables, the analysis can isolate the unique influence of abusive supervision and its attributions on the focal outcome variables of interest, and reduce the risk of confounding effects.

### 3.3. Preliminary Test

#### 3.3.1. Convergent and Discriminant Validity

We employed structural equation modeling (SEM) in Mplus 8 (Muthén & Muthén, 2017) to test the hypotheses. Specifically, confirmatory factor analysis (CFA) was executed to assess the validity of the focal variables and path analysis to test the overall structural model. We conducted CFA on the proposed four-factor model in order to evaluate the convergent and discriminant validity of the measurement model. Since we had a relatively small sample size and a large scale for the measure of abusive supervision (15 items), we created two-item parcels for abusive supervision according to odd and even items (Little et al., 2002). Other variables (performance-promotion attribution, injury-initiation attribution, emotional exhaustion) were represented by the original items. The hypothesized four-factor model yielded a good fit to the data (χ^2^ = 250.37, df = 113, CFI = 0.97, TLI = 0.96, RMSEA = 0.07, SRMR = 0.05). Next, we compared the hypothesized four-factor model with a series of alternative, more parsimonious models. The fit indices of these alternative models are significantly worse than our hypothesized four-factor model (See Table 1). Therefore, these results showcase the discriminant and convergent validity of the measurement model.

#### 3.3.2. Aggregation

Due to the potential multilevel facets of abusive supervision (Priesemuth et al., 2014), it is important to justify the individual nature of the study on abusive supervision. As employees were nested within workgroups led by the same supervisor, abusive supervision perceptions could plausibly show shared and group-level clustering. Therefore, we examined whether there were potential between-group variance and reliable group means to justify aggregating to the group-level of analysis. Referring to the aggregation indices for the focal variables, it indicates how much variance can be explained by group-level factors reflected by the ICC(1), and the reliability of aggregation reflected by the ICC(2). The ICC(1) of the focal variables ranged from 0.03 to 0.09. According to LeBreton and Senter (2008), an ICC(1) ranging from 0.01 to 0.10 represents a tiny amount of variance which is attributed to a group-level effect. The ICC(2) which ranged from 0.16 to 0.37 were also much below the recommended cutoff values of 0.5, indicating that the group means were not sufficiently reliable to support aggregation (LeBreton & Senter, 2008). Accordingly, we treated the constructs as individual-level variables and did not aggregate them to the group level.

#### 3.3.3. Correlations and Control Variables

Table 2 presents the means, standard deviations (SD), and correlation coefficients of the variables, as well as their internal consistency reliability. Abusive supervision is positively correlated with both performance-promotion and injury-initiation attributions. In addition, emotional exhaustion is also positively associated with all focal variables including abusive supervision, performance-promotion attribution, and injury-initiation attribution. Contrary to earlier findings (Y. Zhang & Bednall, 2016), we detected no associations between gender, age, or time working with the supervisor, and the study’s focal constructs, aside from a modest negative link between gender and performance-promotion attribution. We further assessed multicollinearity among all predictors. Specifically, we conducted collinearity diagnostics based on the full regression specification including the main effects, both interaction terms (abusive supervision × performance-promotion attribution and abusive supervision × injury-initiation attribution), and all control variables. The results indicated low multicollinearity: VIFs ranged from 1.14 to 2.70 (all below 5), and tolerance values ranged from 0.38 to 0.93 (all above 0.20), suggesting that multicollinearity is unlikely to materially bias the regression estimates.

#### 3.3.4. Analytic Strategy of the Hypothesis Tests

With the time-lagged and individual nature of the data, we employed path analyses to test the proposed hypotheses using Mplus 8 (Muthén & Muthén, 2017). Due to the small sample size (N = 224) nested in 37 work groups and the use of large scales (e.g., 15-item abusive supervision), path analysis which focuses on mean scores of variables is more suitable than modeling latent variables (Little et al., 2002). As the focal relationships were conceptualized at the individual level and preliminary analyses suggested limited between-group variance, we estimated the proposed model using single-level structural equation modeling (SEM). Based on the two-moderator model, stepwise SEM was adopted to systematically test our hypotheses and evaluate the moderating effects of the attributions. To test the moderating effects, abusive supervision, performance-promotion attribution, and injury-initiation attribution were mean-centered prior to creating the interaction terms (i.e., abusive supervision × performance-promotion attribution and abusive supervision × injury-initiation attribution). Specifically, model 1 included abusive supervision, emotional exhaustion, and control variables as the baseline model for the main effect. Model 2 further included performance-promotion attribution and injury-initiation attribution. Model 3 was the full model including all focal variables and the two interaction terms. The strategy allows us to (1) verify the consistency of the main effect across models, (2) determine the unique contribution of each moderator, and (3) detect potential suppression or collinearity effects when both moderators are included.

## 4. Results

The results of all the proposed hypotheses are presented in Table 3. Our analysis involved all the control variables of gender, age, and time working with supervisors in the main models. The results demonstrate no significant impacts of control variables on the outcomes. Thus, the hypothesized relationships account for the primary variance observed.

H1 predicts that abusive supervision is positively associated with emotional exhaustion. The results of Model 1 showed a significant positive relationship between abusive supervision and emotional exhaustion (*b* = 0.56, *p* < 0.001; 95%CI [0.40, 0.71]). This result remained unchanged (*b* = 0.42, *p* < 0.001; 95%CI [0.22, 0.62]) in Model 2. Neither performance-promotion attribution nor injury-initiation attribution were significantly associated with emotional exhaustion, indicating that the effect of abusive supervision on emotional exhaustion was robust; thus, H1 is supported.

H2 predicts the buffering effect of performance-promotion attribution. The results of model 3 revealed a positive and significant interaction (*b* = 0.14; *p* < 0.01; 95%CI [0.05, 0.22]), whereas the main effect of abusive supervision became non-significant. Accordingly, the significant interaction indicated that association between abusive supervision and emotional exhaustion depended on the level of performance-promotion attribution. Contrary to H2, the positive interaction suggested an exacerbating rather than buffering effect; thus, H2 is not supported yet the exacerbating effect of performance-promotion attribution is supported.

**Figure 2:**
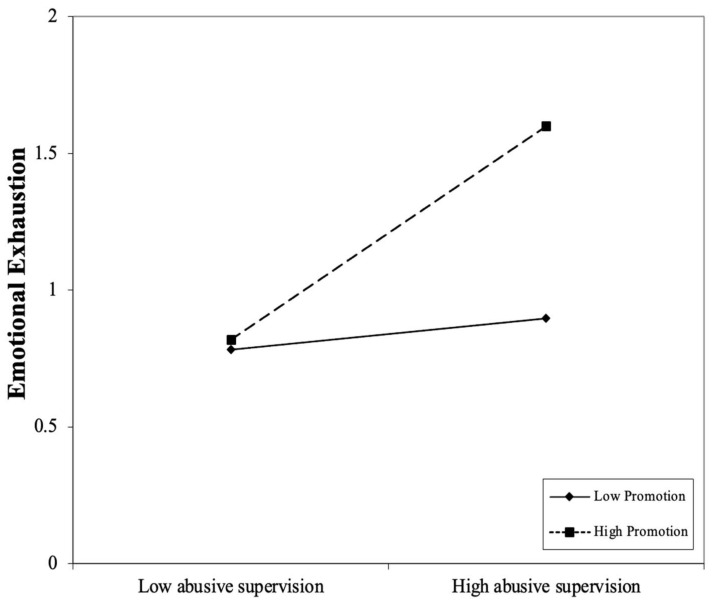
The moderating effect of performance-promotion on the relationship between abusive supervision and subordinate emotional exhaustion.

To further probe this interaction, we conducted a simple slope analysis at three levels of performance-promotion attribution: Low (−1 SD), mean, and high (+1 SD). The conditional effect of abusive supervision on emotional exhaustion increased as performance-promotion attribution increased, with slope estimates of *b* = 0.09 (*p* = 0.64) at one standard deviation below the mean, *b* = 0.25 (*p* = 0.27) at the mean, and *b* = 0.42 (*p* = 0.13) at one standard deviation above the mean. Although none of these conditional effects reached statistical significance, the increasing slope estimates and decreasing *p* values were descriptively consistent with the positive interaction effect (*b* = 0.14; *p* < 0.01; 95%CI [0.05, 0.22]) observed in Model 3. According to the results, the interaction pattern is depicted in Figure 2.

H3 predicts the moderating effect of injury-initiation attribution. The results of Model 3 revealed a non-significant interaction (*b* = 0.02, *p* = 0.79; 95%CI [−0.08, 0.11]); thus, H3 is not supported.

Due to the nested nature of the data, we conducted an additional robustness check using multilevel SEM. Specifically, we re-estimated the full model using a random-intercept two-level specification that accounted for between-group clustering while retaining the focal relationships at the individual level (Preacher et al., 2010). The pattern of results was unchanged (e.g., the moderating effect is significant *b* = 0.14, *p* < 0.01, 95% CI [0.06, 0.22]), indicating that the results were robust.

## 5. Discussion

The present study investigated the moderating role of performance-promotion and injury-initiation attributions in the relationship between supervision and emotional exhaustion. The findings affirm that abusive supervision is positively associated with emotional exhaustion. However, contrary to expectations, performance-promotion attribution exacerbates, rather than buffers, the positive association between abusive supervision and emotional exhaustion. Furthermore, the results indicate that the moderating effect of injury-initiation attribution is not significant. The following sections elaborate on the theoretical implications of these findings and outline their practical relevance.

### 5.1. Theoretical Implication

Prior studies found an interactive influence between abusive supervision and performance-promotion attribution on employee performance outcomes such as creativity (Liu et al., 2012), supervisor-rated OCB (Yu & Duffy, 2021), and work engagement (Huh & Lee, 2022). Nevertheless, how these attributions moderate the relationship between abusive supervision and employees’ health-related well-being has not been subject to empirical scrutiny. To address this void, the present study represents an important contribution by examining whether similar moderating effects of the attributions extend to subordinate emotional exhaustion. Aligning with past literature (Aryee et al., 2008), the findings support the main effect of abusive supervision on emotional exhaustion, reaffirming the detrimental effect of abusive supervision on employee well-being.

Contrary to expectations, the findings reveal that performance-promotion attribution amplifies, rather than buffers, the influence of abusive supervision on emotional exhaustion. Drawing on COR theory (Hobfoll, 1989), existing studies identified performance-promotion attribution as a potential individual resource that can buffer the negative influence of abusive supervision on the performance outcomes of creativity (Liu et al., 2012), task performance (Yang et al., 2020), and OCB (Yu & Duffy, 2021). However, when it comes to the employee well-being outcome of emotional exhaustion, the present study reveals a converse perspective. That is, performance-promotion attribution exacerbates the positive relationship between abusive supervision and emotional exhaustion. This effect likely arises because performance-promotion attribution stimulates individuals to persist in striving for higher performance standards under abusive supervision, thereby emanating working pressure and emotional burden. In this regard, while performance-promotion attribution can help sustain subordinate performance (Huh & Lee, 2022; Kim et al., 2019; Yu & Duffy, 2021), it might expose a “double edge” when individual well-being is taken into account.

Specifically, performance-promotion attribution is an instrumental resource, motivating subordinates to improve performance, but it simultaneously functions as a job demand imposing higher performance requirements on subordinates. To preserve self-worth and avoid further criticism, subordinates tend to invest additional resources to close presumed performance gaps, monitor their output more vigilantly, and display visible improvement to signal compliance. Each of these self-regulatory acts further consume individual resources including energetic, affective, and temporal resources while possibly leaving the original source of the demands unchanged (i.e., perception of leaders’ abusive behaviors). Consistent with COR theory, this logic implies a boundary condition within which the exacerbating effect may manifest or be stronger when employees have limited replenishing resources or when performance demands are chronic and highly salient (e.g., high workload and time pressure), because sustained self-regulatory investment is more likely to outstrip available resources and trigger loss spirals. Performance-promotion attribution thus transforms abusive supervision from a finite stressor into an open-ended resource drain. In this regard, the “positive” attribution might compel abused subordinates to invest continuous and substantial resources into enhancing their performance (Liu et al., 2012; Yang et al., 2020; Yu & Duffy, 2021), potentially causing elevated emotional exhaustion as the side-effect of improving performance.

Based on COR theory (Hobfoll, 1989), the findings indicate that seemingly “benign” and “motive” attributions (e.g., correction, warning, and motivation) may also operate as a job demand, undermining individual well-being, particularly when the heightened performance expectations and effort requirements they impose surpass employees’ available resources. Though performance-promotion attribution may alleviate the demands of abusive supervision by framing it as performance-oriented and helping subordinates make sense of harsh treatment as goal-directed feedback, its instrumental value often comes at the cost of consuming subordinates’ emotional and physical resources. Therefore, performance-promotion attribution may not constitute a personal resource as previously suggested (Liu et al., 2012; Yang et al., 2020; Yu & Duffy, 2021), given that safeguarding employee well-being is a core organizational priority, and when compromised, could ultimately undermine sustainable performance and organizational benefits. Indeed, although positive attributions may stimulate performance enhancement in the short term, their benefits are likely to be transient once the potential demands to heightened emotional exhaustion are taken into account.

On the other hand, the study suggested that the moderating effect of injury-initiation attribution is nonsignificant, a result that diverges from the trend of existing evidence (Liu et al., 2012; Yu & Duffy, 2021) and suggests an alternative possibility regarding the negative attributions of abusive supervision. Drawing on attribution theory (Heider, 1958), a plausible explanation could be that the effect of negative attributions may be trivial because abusive supervision already carries a default presumption of malevolent intent (i.e., abusive supervision is widely viewed as unethical and harmful). In this manner, labeling abusive supervision explicitly as injury-initiation intent could add little incremental variance to an appraisal structure that is close to a threat ceiling. Unlike performance-promotion attribution, which may provide subordinates with a psychologically functional rationale that activates sensemaking processes rather than resignation, preserves goal-related meaning, and sustains effort despite mistreatment, subordinates who endorse injury-initiation attributions may view abusive supervision as an expected feature of authority rather than a diagnostically informative signal about intent. Therefore, as subordinates have already coded the leaders’ abusive behaviors as gratuitous harm, an additional negative attribution is not likely to intensify the demands on well-being outcomes (i.e., emotional exhaustion). This saturation effect aligns with attribution theory’s principle of discounting: When one highly plausible cause (i.e., malice of abusive supervision) explains an event, alternative causes contribute negligible explanatory power (Kelley, 1973). The perspective suggests that attribution valence operates on a threshold rather than a linear scale (Kelley, 1973). Thus, once a subordinate crosses the threshold into certainty of malevolent intent, further nuance becomes psychologically moot.

Moreover, examining injury-initiation attribution in a Chinese context (e.g., a state-owned and high power-distance company) is theoretically and practically important because supervisory behaviors may be interpreted differently in other settings (US or European organizations). By highlighting that injury-initiation attribution might offer limited incremental explanatory value in Chinese traditional industries, the results imply that the malevolent intent of abusive supervisory behaviors is often presumed and directly exerts a negative influence. Specifically, Chinese traditional industries, characterized by centralization, high power-distance, and stronger norms of hierarchical control, may largely normalize harsh supervisory conduct, and constrain employees’ discretion to reinterpret or contest it. As a result, injury-initiation sensemaking could be relatively uniform (i.e., “already assumed”), leaving little variance for injury-initiation attributions to function as a moderator. Drawing on attribution theory, this suggests a theoretically derived boundary condition within which injury-initiation attribution should be more likely to operate as a meaningful moderator when intents of abusive supervision are relatively ambiguous and socially contestable (e.g., European workplace settings characterized by lower power-distance and weaker hierarchical control norms). In such settings, employees have greater latitude to interpret supervisory intent, which could amplify variability in negative sensemaking and heighten resource-loss appraisals, thereby strengthening the moderating role of injury-initiation attribution.

In sum, the findings advance the abusive supervision literature by complementing existing theorizing on both performance-promotion and injury-initiation attributions (Kim et al., 2019; Liu et al., 2012; Yu & Duffy, 2021) with a more nuanced understanding. Drawing on attribution theory (Heider, 1958) and COR theory (Hobfoll, 1989), the empirical findings suggest that performance-promotion attribution may exacerbate, rather than buffer, the association between abusive supervision and emotional exhaustion, whereas injury-initiation attribution may not exhibit a reliable moderating effect.

### 5.2. Practical Implications

Scholars and practitioners emphasized the intent behind leaders’ aggressive behaviors, such as promoting performance and halting obvious mistakes, which might potentially justify these negative supervisory behaviors (Liu et al., 2012; Tepper, 2000, 2007). However, the present study suggests caution in endorsing these so-called “tough love” rationales and highlights the importance of reducing abusive supervision or developing other effective coping strategies to mitigate their harmful effects on subordinates. The reason is that performance-promotion attribution may not buffer, but rather exacerbates, the negative impact of abusive supervision on individual well-being. Despite evidence that positive attributions for abusive supervision may help employees maintain task performance (Yang et al., 2020; Yu & Duffy, 2021), encouraging employees to reinterpret abusive behaviors as developmental remains difficult to sustain, and could raise concerns about humanity and ethical acceptability. It is highly recommended to equip leaders and managers with evidence-based feedback techniques that combine supervisory behavioral norms and subordinates’ psychological safety. Leadership-development curricula can integrate role-play scenarios demonstrating how ostensibly motivational criticism and aggressiveness drain subordinates’ emotional resources when unaccompanied by tangible support (e.g., communication, coaching time, and autonomy). By foregrounding behavioral change and resource replenishment, rather than cognitive reframing, organizations can reduce the likelihood that “performance-promotion” narratives mask the harm of leaders’ aggressive behaviors. Meanwhile, the implementation of stringent regulations is also necessary to compel leaders to adhere to ethical standards and behavioral norms, thereby achieving active and passive reductions in abusive supervision in the workplace.

### 5.3. Limitations and Future Directions

This study contains several limitations that should be addressed in future research. First, although our time-lagged and nested data from a Chinese company has strengths (L. H. Liang et al., 2016; Wang et al., 2020), some shortcomings remain. Our conceptual model includes multiple hypotheses and several multi-item constructs, yet is tested with a relatively small sample size (N = 224) nested within 37 groups from a single organization. While the analytic procedures are appropriate and justified for the data at hand, a larger sample size would allow a comprehensive test of the model (e.g., full SEM with latent variables). In addition, another limitation is that all focal variables were collected from employees. Although the time-lagged design helps to mitigate this concern and the focal variables are internal perceptions that are well suited to self-report surveys, common method bias cannot be entirely ruled out. Future research should therefore employ multi-source designs (e.g., supervisor ratings and archival performance indicators) and, where feasible, adopt experimental or longitudinal field designs to address common method bias more rigorously.

Second, the research was conducted in a single Chinese company, which may limit the generalizability of the findings to other organizational and cultural contexts. Environmental factors can shape employees’ reactions to abusive supervision and its attributions (Tepper, 2007; Tepper et al., 2017). Importantly, as noted above, organizational, industrial, and national characteristics might significantly shape the strength of moderating effects of attributions (Hobfoll, 1989; Hobfoll et al., 2018). For example, some employees are desensitized to supervisory behaviors and perceived harmful intents, while others may experience stronger cognitive and emotional reactions. A frequently cited case is military settings, where harsh treatment may coexist with high effectiveness. Future research should therefore draw on cross-cultural and multi-organization samples to enhance external validity and assess the robustness of these findings across different cultural and occupational contexts.

Third, although our findings raise a notion that abusive supervision attributions might not function as the presumed patterns (Liu et al., 2012), this line of inquiry remains nascent, as mentioned in single the Chinese company. Based on our best knowledge, the counterintuitive amplification effect of performance-promotion attribution has been documented only for emotional exhaustion. It is unclear whether the same pattern can be duplicated across other critical outcomes such as job satisfaction, organizational commitment, or turnover intentions. Future research should therefore adopt a multi-criterion lens to test whether performance-promotion attribution consistently exacerbates (or perhaps mitigates) a broader array of attitudinal and behavioral consequences. The similar line of inquiry is also warranted for injury-initiation attribution. Such evidence would further clarify the functions of these attributions. In addition, prior work has focused primarily on abusive supervision attributions as moderators, overlooking the antecedents that cultivate these cognitive frames (Liu et al., 2012; Yu & Duffy, 2021). It shall be promising to examine whether organizational factors such as leader credibility, organizational justice climates, or individual dispositions can predispose subordinates toward performance-promotion or injury-initiation attributions. Identifying these drivers would move the literature beyond “whether” attributions matter to “why” they emerge, thereby opening actionable avenues for prevention and strengthening employees’ sense of control.

## 6. Conclusions

Attributions may be crucial to understanding abusive supervision because they represent a key interpretive mechanism that structures how subordinates make sense of the behavior and respond to it. The present study articulates the role of individual attributions in the relationship between abusive supervision and individual well-being. Given the fundamentally harmful nature of abusive supervision, its detrimental effects on well-being are likely to remain dominant, leaving limited scope for attributions to mitigate them. We advocate inhibiting abusive supervision, and meanwhile providing effective coping solutions by achieving a comprehensive understanding of abusive supervision.

## Figures and Tables

**Table 1:** Measurement models and fit indices.

	Χ^2^	df	CFI	TLI	RMSEA	SRMR
Hypothesized four-factor model	250.37	113	0.97	0.96	0.07	0.05
Three-factor model (combining abusive supervision and injury-initiation attribution)	736.76	116	0.85	0.82	0.16	0.07
Three-factor model (combining performance-promotion and injury-initiation attributions)	2019.54	116	0.53	0.45	0.27	0.27
Two-factor model (combining abusive supervision and its attributions)	1369.85	118	0.69	0.64	0.22	0.17
One-factor model	2206.31	123	0.48	0.43	0.28	0.27

Note. N = 224, CFI = comparative fit index, TLI = Tucker–Lewis index, RMSEA = root mean square error of approximation, SRMR = standardized root mean square residual.

**Table 2:** Bivariate Correlations.

Variable	Mean	Std Deviation	1	2	3	4	5	6	7
1. AS	1.54	0.81	(0.97)						
2. Promotion	3.95	1.22	0.14 *	(0.89)					
3. Injury	1.70	1.16	0.64 **	0.13	(0.91)				
4. Exhaustion	3.06	1.13	0.45 **	0.20 **	0.40 **	(0.92)			
5. Gender	1.31	0.46	−0.08	−0.22 **	−0.10	−0.05	(1)		
6. Age	42.93	8.97	0.16 *	0.01	0.16 *	0.03	−0.22 **	(1)	
7. Time with Supervisor	9.98	8.62	0.13 *	0.06	0.10	0.04	−0.16 *	0.48 **	(1)

Note. N = 224 subordinates. AS = abusive supervision; Promotion = performance-promotion attribution; Injury = injury-initiation attribution. Reliabilities are reported in parentheses on the diagonal. * *p* < 0.05; ** *p* < 0.01 (all tests are two-tailed).

**Table 3:** The Main and Interactive Effects of Abusive Supervision and Attributions on Subordinate Emotional Exhaustion.

Variables	Model 1		Model 2		Model 3	
	*b*	*SE*	*b*	*SE*	*b*	*SE*
Independent						
Abusive supervision	0.56 ***	0.08	0.42 ***	0.12	−0.27	0.18
Performance promotion			0.11	0.06	−0.09	0.11
Injury-initiation			0.13	0.10	0.12	0.12
Abusive supervision × performance-promotion					0.14 **	0.05
Abusive supervision × injury-initiation					0.02	0.06
Demographic (Control)						
Gender	−0.08	0.15	−0.01	0.15	0.01	0.14
Age	−0.01	0.01	−0.01	0.01	−0.01	0.02
Time with supervisor	−0.01	0.01	−0.01	0.01	−0.01	0.02

Note. N = 224 subordinates. Unstandardized model coefficients are presented. All significance tests are two-tailed. * *p* < 0.05; ** *p* < 0.01; *** *p* < 0.001.

## Data Availability

The data that support the findings of this study are available from the corresponding author upon reasonable request.

## Appendix A. Measurement Items

Abusive Supervision (Tepper, 2000)

1. My supervisor ridicules me.
2. My supervisor tells me my thoughts or feelings are stupid.
3. My supervisor gives me the silent treatment.
4. My supervisor puts me down in front of others.
5. My supervisor invades my privacy.
6. My supervisor reminds me of my past mistakes and failures.
7. My supervisor does not give me credit for jobs requiring a lot of effort.
8. My supervisor blames me to save himself/herself embarrassment.
9. My supervisor breaks promises he/she makes.
10. My supervisor expresses anger at me when he/she is mad for another reason.
11. My supervisor makes negative comments about me to others.
12. My supervisor is rude to me.
13. My supervisor does not allow me to interact with my coworkers.
14. My supervisor tells me I am incompetent.
15. My supervisor lies to me.

Attributions of Abusive Supervision (Liu et al., 2012)

The survey read as follows: “Abusive supervision means the sustained display of hostile, verbal and non-verbal behaviors, excluding physical contact. To what extent, do you agree that the following may be the reason for or cause of your supervisor’s behaviors toward you, which could be considered abusive?”

Performance-Promotion Motives

1. Desire to elicit high performance from me.
2. Desire to send me messages that mistakes will not be tolerated.
3. Desire to alert me of my mistakes and problems.
4. Desire to push me to work harder.
5. Desire to stimulate me to meet my performance goals.

Injury-Initiation Motives

1. Desire to cause injury on me.
2. Desire to hurt my feelings.
3. Desire to harm my reputation.
4. Desire to make me feel bad about myself.
5. Desire to sabotage me at work.

Emotional Exhaustion (Maslach et al., 1996)

1. I feel emotionally drained from my work.
2. I feel used up at the end of the workday.
3. I feel tired when I get up in the morning and have to face another day on the job.
4. Working all day is really a strain for me.
5. I feel burned out from my work.
